# Integrating BSA-Seq, QTL Mapping, and RNA-Seq to Identify Candidate Genes for Hollow Heart in Cucumber Fruits

**DOI:** 10.3390/plants15091299

**Published:** 2026-04-23

**Authors:** Mengyao Kong, Chenran Gu, Xiaoyue Li, Yanwen Yuan, Jiaxi Li, Zhiwei Qin, Ming Xin

**Affiliations:** Key Laboratory of Biology and Genetic Improvement of Horticultural Crops (Northeast Region), College of Horticulture and Landscape Architecture, Northeast Agricultural University, Harbin 150030, China; kongmengyao2024@163.com (M.K.);

**Keywords:** cucumber, hollow heart, BSA-seq, QTL mapping, RNA-seq

## Abstract

Cucumber (*Cucumis sativus* L.) is a globally significant vegetable crop, and its fruit quality remains a major focus of research. The hollow-heart trait, characterized by internal cracks or cavities, severely compromises both the commercial value and edible quality of cucumber fruit. In this study, a six-generation segregating population (P_1_, P_2_, F_1_, F_2_, BC_1_P_1_, BC_1_P_2_) was developed from the parental lines “JZ6-1-2” and “D0432-3-4”. BSA-seq was employed to map candidate genomic regions associated with the hollow-heart trait to chromosomes 2, 3, and 7. Subsequently, a major QTL for the trait was delineated on chromosome 7, spanning a region containing 98 genes. Comparative RNA-seq between the parental lines identified 2141 differentially expressed genes. The integration of QTL mapping and RNA-seq data revealed 11 candidate genes residing within the key QTL interval. Through further validation via qRT-PCR, gene sequence comparison, and gene annotation, *Csa7G039280* was identified as a promising candidate gene regulating hollow-heart formation, potentially via the lignin biosynthesis pathway. The identification of these candidate regions and genes provides critical information for molecular breeding aimed at developing non-hollow-heart cucumber varieties, thereby enhancing the understanding of the genetic regulatory mechanisms underlying this economically important trait.

## 1. Introduction

Fruits are vital organs of horticultural crops, developing from the ovary or other floral parts (such as the receptacle and sepals) after pollination and fertilization of the pistil [1]. The hollow heart phenomenon in fruits is characterized by the formation of internal cracks or cavities, which arise from differential expansion rates between cells in the locule region and those in the surrounding pericarp tissue [2,3]. In cucumber (*Cucumis sativus* L.), this phenomenon manifests as carpel separation, where fused carpels detach along the suture line [4]. Based on cytological observations, certain cucumber cultivars, such as “Monastyrski” have been reported to exhibit increased susceptibility to fruit hollow heart under prolonged environmental stress, including high soil nitrogen, water deficit, and abrupt temperature fluctuations during fruit development [5].

Although the development of hollow heart in cucumber fruit is strongly influenced by environmental factors, it remains a heritable trait under genetic control [6]. Quantitative trait loci (QTL) mapping has been conducted using segregating populations derived from Sikkim-type cucumber inbred lines. In a recombinant inbred line (RIL) population (WI7088D × Coolgreen), three QTLs (*mfh1.1*, *mfh2.1*, *mfh3.1*) associated with fruit hollow heart were identified, whereas one QTL (*mfh2.1*) was detected in an F_2:3_ population (WI7120 × 9930) [7]. Subsequent fine-mapping of the *mfh2.1* locus by Shang et al., using the non-hollow line “9930” and hollow line “WI7120”, delineated a candidate interval on chromosome 2 (18966421-19017345 bp) containing nine genes. Combined qRT-PCR and gene functional analysis revealed significantly differential expression of *CsRPT4Bb* between the parental lines. Since *CsRPT4Bb* is implicated in rapid cell expansion during early fruit development, it was proposed as a candidate regulator of hollow heart, though functional validation remains pending [8]. In parallel, Zhou et al. proposed a cellular mechanism for hollow heart formation, suggesting that it results from the separation of a zipper-like double-cell layer in the carpel transport channel during locule expansion. Through bulk segregant analysis sequencing (BSA-seq) and kompetitive allele-specific PCR (KASP) genotyping, the same study identified *CsALMT2* (aluminum-activated malate transporter 2), located on chromosome 1 (25185078–25203154 bp), as a candidate gene. Downregulation of *CsALMT2* in hollow fruits suggested a potential negative regulatory role in the trait [4]. Nevertheless, the precise molecular mechanisms and regulatory networks underlying hollow heart formation remain to be fully elucidated.

From another perspective, comparative transcriptome sequencing by Li et al. indicated that diminished expression and biosynthesis of lignin, cellulose, and hemicellulose impair cell wall integrity, thereby contributing to fruit hollow heart [9]. Correspondingly, transcriptomic analyses conducted by Zhou et al. underscored the pivotal role of the phenylpropanoid biosynthesis pathway in the formation of hollow hearts in cucumber fruits. Their findings revealed pronounced differences in lignin accumulation between hollow and non-hollow cucumber varieties, suggesting that elevated lignin levels may promote the development of the hollow heart trait [10].

*CsGID1a*, a gibberellin receptor gene, plays a crucial role in ovary development during cucumber fruit formation. Its silencing has been shown to induce plant dwarfism and impair locule development [11]. Moreover, two bHLH transcription factors, *CsSPT* (*SPATULA*) and *CsALC* (*ALCATRAZ*), regulate the expression of genes involved in transmitting tract development, auxin-mediated signaling, and cell wall organization. These factors function as both homodimers and heterodimers to modulate fruit cavity formation and influence female fertility [12]. Given that the hollow heart trait in cucumber fruit also exhibits characteristics associated with developmental sterility, it is hypothesized that additional regulatory pathways may be involved in its formation.

Both BSA-seq and QTL mapping primarily focus on genomic variation; they delineate chromosomal regions related to target traits and propose potential candidate genes based on positional information, yet often lack direct evidence linking gene expression levels to phenotypic outcomes. In contrast, RNA sequencing (RNA-seq) serves as a key approach for profiling gene expression. By extracting total RNA from samples and determining transcript sequences, RNA-seq provides comprehensive and quantitative information on transcript abundance and differential expression [13,14]. Therefore, the integration of BSA-seq, QTL mapping, and RNA-seq has emerged as an effective strategy for efficiently dissecting target genomic regions and rapidly screening functional genes associated with agronomic traits. This integrated approach has been widely applied in studying key traits across various plant species, as exemplified by the identification of candidate genes involved in petiole color in papaya [15], flesh firmness in apple [16], and storability in Dongxiang wild rice [17].

Cucumber is a globally important vegetable crop, but its production is severely constrained by fruit hollow heart, which drastically reduces fruit quality and causes substantial economic losses. Addressing this pressing issue requires the genetic improvement of cultivars, for which the identification of key regulatory genes is a fundamental prerequisite. In this study, the hollow-heart-susceptible inbred line “JZ6-1-2” was crossed with a resistant line “D0432-3-4” to develop a six-generation segregating population, and an integrated strategy combining BSA-seq, QTL mapping, and RNA-seq was used to identify the most promising candidate gene underlying fruit hollow heart in cucumber. The results provide a clearer understanding of the genetic basis of cucumber fruit hollow heart and lay a foundation for the molecular breeding of hollow-heart-resistant cucumber varieties.

## 2. Results

### 2.1. Genetic Analysis of Hollow Heart in Cucumber Fruits

**Figure 1:**
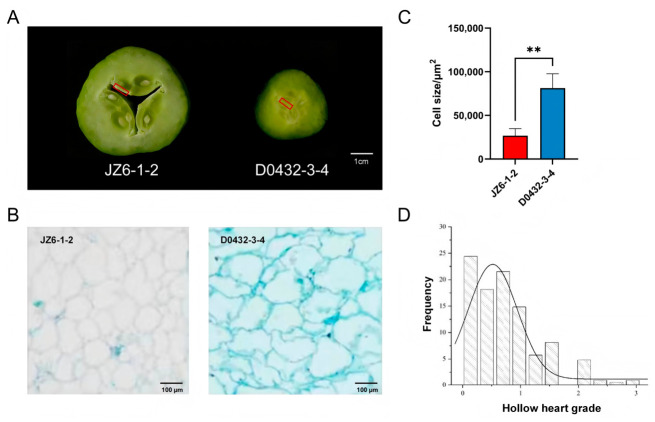
Phenotypes of cucumber varieties and frequency distribution of hollow heart traits in the F_2_ population. (**A**) Schematic diagrams of the cross-sections in the middle of fruits on the ninth day after female flower opening for the hollow-heart cucumber variety “JZ6-1-2” and the non-hollow-heart cucumber variety “D0432-3-4”; the red boxes indicate the carpel suture region from which tissue samples were collected for paraffin sectioning and RNA-seq; (**B**) Paraffin cross-sections of the carpel region of “JZ6-1-2” and “D0432-3-4” at 9 DPA. Bar = 100 μm; (**C**) Statistical diagram of cell area. **, *p* < 0.01; (**D**) Histogram of the frequency distribution of hollow heart traits in the F_2_ population. The hollow heart grade was scored on a 0–4 scale based on the severity of cavity formation: 0, no hollow heart; 1, slight (0–1% of cross-sectional area); 2, mild (1–10%); 3, moderate (10–40%); 4, severe (≥40%).

According to the cavity evaluation of cucumber germplasm resources conducted by Qin et al., the South China-type cucumber variety “JZ6-1-2” was identified as a hollow-heart variety at 9 days post-anthesis (9 DPA), with the hollow area accounting for 10% to 40% of the cross-sectional area, indicating a pronounced cavity. In contrast, the European greenhouse-ecotype cucumber variety “D0432-3-4” did not exhibit any hollow heart phenomenon at 9 DPA (Figure 1A) [18]. Paraffin section observations on cross-sections of the carpel region of cucumber fruits revealed that the cell size in the carpel region of hollow cucumber cultivars was significantly smaller than that in non-hollow cultivars (*p* < 0.01), suggesting a significant correlation between carpel cell size and fruit hollow heart formation (Figure 1B,C).

To identify genetic loci associated with hollow heart in cucumber fruit, a six-generation population (P_1_, P_2_, F_1_, F_2_, BC_1_P_1_, BC_1_P_2_) was developed from a cross between the hollow heart-susceptible line “JZ6-1-2” (female parent) and the non-hollow heart line “D0432-3-4” (male parent). The F_1_ plants displayed an intermediate hollow heart phenotype relative to the two parents (Table 1). Backcross populations showed expected phenotypic tendencies: BC_1_P_1_ plants were predominantly hollow, whereas BC_1_P_2_ plants largely exhibited the non-hollow phenotype (Table 1). In the F_2_ population, the hollow heart trait displayed continuous phenotypic variation with a skewed distribution, consistent with quantitative inheritance (Figure 1D).

### 2.2. Identification for Candidate Intervals Through BSA-Seq and QTL Mapping

To refine the genetic mapping of the hollow heart trait in cucumber, BSA-seq was performed using four DNA pools: the two parental bulks (HLP1, HLP2, each consisting of 25 individuals) and the phenotype-based bulks for hollow (HL) and non-hollow (NHL) phenotypes, where the HL bulk consisted of 25 F_2_ individuals with a hollow heart grade of 3, and the NHL bulk consisted of 25 F_2_ individuals with a grade of 0. After quality filtering, a total of 28.37 Gb of clean data were obtained. The Q30 scores ranged from 94.55% to 95.09%, and GC content varied between 36.28% and 37.06% (Table S1). Alignment of the sequencing reads to the Chinese Long v2 reference genome showed that over 92% of reads were mapped, with properly paired reads exceeding 73% for all four samples (Table S2). The average sequencing depth across the genome was 31.25×, and more than 97% of the genome was covered at a depth of at least 5× (Table S3). These metrics confirm that the sequencing data were of high quality and suitable for subsequent analysis.

**Figure 2:**
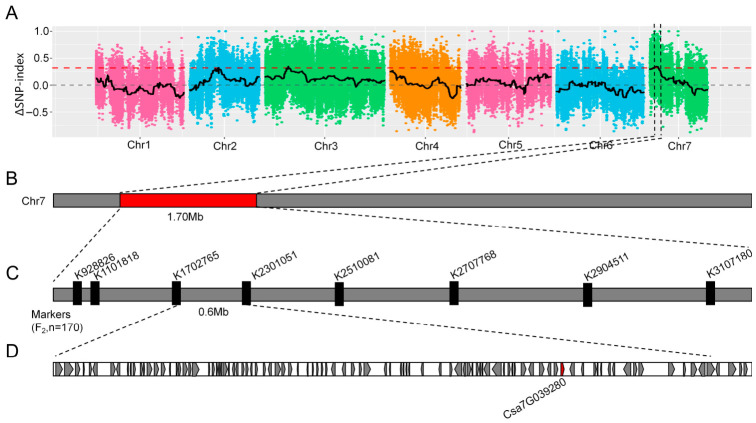
Mapping of genes related to hollow heart in cucumber fruits. (**A**) Distribution of SNP-index association values across chromosomes. Colored dots represent ΔSNP-index values, while the black line indicates the fitted ΔSNP-index. The red line denotes the threshold line at the 99th percentile; (**B**) The red box indicates the approximate location of the major QTL in the middle region of chromosome 7 in cucumber; (**C**) Distribution of KASP markers within the approximate localization interval of the target gene; (**D**) Ninety-eight candidate genes are located between the two molecular markers, K1702765 and K2301051.

SNP-index association analysis was performed using 25813 high-quality SNPs derived from bulks. No genomic region was significantly associated with the trait when a confidence level of 0.99 was applied to the ΔSNP-index. To identify potential candidate regions, the threshold was lowered to the 99th percentile of the fitted ΔSNP-index, 0.32, and three candidate intervals were detected on chromosomes 2, 3, and 7 (Figure 2A). Among these, an interval on chromosome 2 (9570000–9590000, 0.02 Mb) contained one gene, an interval on chromosome 3 (7630000–7790000, 0.16 Mb) contained 25 genes, and an interval on chromosome 7 (1200000–2900000, 1.70 Mb) contained 284 genes (Figure 2B).

In order to refine the candidate intervals, KASP markers were developed for genotyping. Screening for polymorphisms identified thirteen KASP primers suitable for linkage analysis, with five located on chromosome 3 and eight on chromosome 7 (Figure S1). These markers were used to genotype 170 F_2_ individuals. Subsequent QTL mapping based on the constructed genetic linkage map revealed no significant locus on chromosome 3, but identified one QTL on chromosome 7 associated with hollow heart (Figure 2C), confirming the reliability of the BSA-seq results. This QTL was delimited between markers K1702765 and K2301051 (Figure S1, Table S4), covering a physical interval of approximately 0.6 Mb and encompassing 98 predicted genes (Figure 2D). Among these, 76 genes have functional annotations, while 22 remain uncharacterized (Table S5).

### 2.3. Transcriptomic Profiling of Hollow Heart in Cucumber Fruits

**Figure 3:**
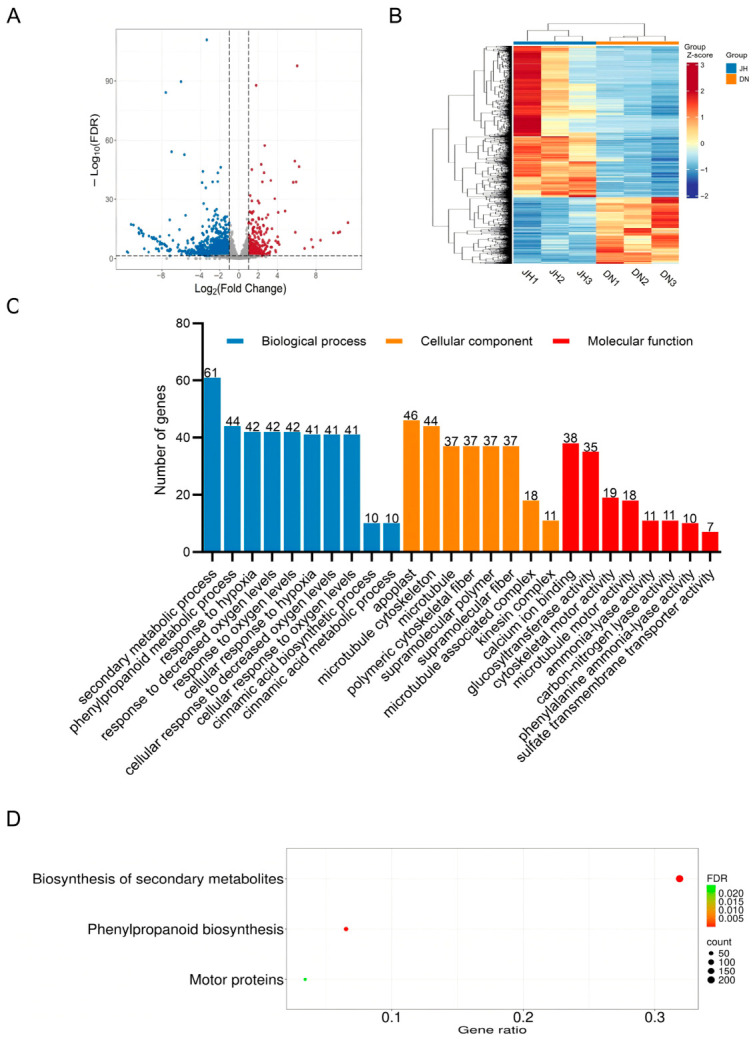
DEGs analysis of RNA-seq. (**A**) Volcano plot of DEGs. Red dots, upregulated DEGs. Blue dots, downregulated DEGs. Gray dots, non-DEGs; (**B**) Heatmap of DEGs; (**C**) Scatter plot of GO enrichment; (**D**) Scatter plot of KEGG enrichment.

For six samples (DN1–DN3, JH1–JH3) collected from the carpel suture region at 9 DPA (indicated by red boxes in Figure 1A), an average of 10.98 Gb of clean RNA-seq data was obtained per sample after quality control, with a mean Q30 score of 97.78% and GC content ranging from 40% to 50% (Table S6). For each library, high-quality filtered reads accounted for >95% of the original sequenced reads (Table S6), confirming the overall high quality of the sequencing data. Furthermore, qRT-PCR validation was performed on six randomly selected differentially expressed genes (DEGs) to verify their expression patterns. The results showed that their relative expression levels were consistent with the corresponding FPKM values from the RNA-seq analysis, and exhibited significant differences between JH and DN (Figure S2), supporting the reliability of the transcriptomic results. A total of 2141 DEGs were identified in the comparison between “D0432-3-4” and “JZ6-1-2”, including 1485 downregulated and 656 upregulated genes (Figure 3A). Hierarchical clustering of all DEGs in a heatmap revealed two distinct expression patterns: one group showed higher expression in DN (upregulated DEGs), while the other exhibited higher expression in JH (downregulated DEGs) (Figure 3B).

Functional enrichment analysis of the differentially expressed genes was performed using Gene Ontology (GO) and Kyoto Encyclopedia of Genes and Genomes (KEGG) databases. In the DN_JH comparison, all DEGs were assigned to 2850 GO terms. These included 1904 terms in the biological process category, 296 in cellular component, and 650 in molecular function. Of the 2850 assigned GO terms, 69 were significantly enriched (FDR < 0.05). These included 53 terms in biological Process, 8 in cellular component, and 8 in molecular function. For clarity, only the top 10 most significantly enriched terms within each category are displayed in Figure 3C. Enriched biological process terms were primarily related to cellular responses and signaling triggered by environmental stimuli (hypoxia, jasmonic acid, and water deficit), broad metabolic processes (especially secondary and phenylalanine metabolism), and core cellular activities such as the cell cycle and division. Within the cellular component, significant terms involved microtubules and associated complexes, cytoskeletal polymers, and the apoplast. Molecular function terms largely encompassed various enzymatic, transporter, binding, and cytoskeletal motor activities. Notably, fewer genes were enriched in pathways including photosynthetic electron transport in photosystem I, positive regulation of auxin-mediated signaling, and sulfate transmembrane transporter activity. In contrast, processes such as cell division, the cell cycle, secondary metabolite metabolism, and response to water were represented by a larger number of genes. These patterns suggest that the formation of hollow heart in cucumber fruit may be associated with the aforementioned biological processes and molecular functions.

In the DN_JH comparison, DEGs were enriched in a total of 121 KEGG pathways, with the threshold set at FDR < 0.05. KEGG analysis revealed significant enrichment in three pathways, including biosynthesis of secondary metabolites, phenylpropanoid biosynthesis, and motor proteins. The pathway with the highest number of enriched genes was biosynthesis of secondary metabolites, leading to the hypothesis that the occurrence of hollow hearts in cucumber fruits may be related to the metabolism and synthesis of secondary metabolites (Figure 3D).

### 2.4. Integrated Analysis of BSA-Seq, QTL Mapping and RNA-Seq

**Figure 4:**
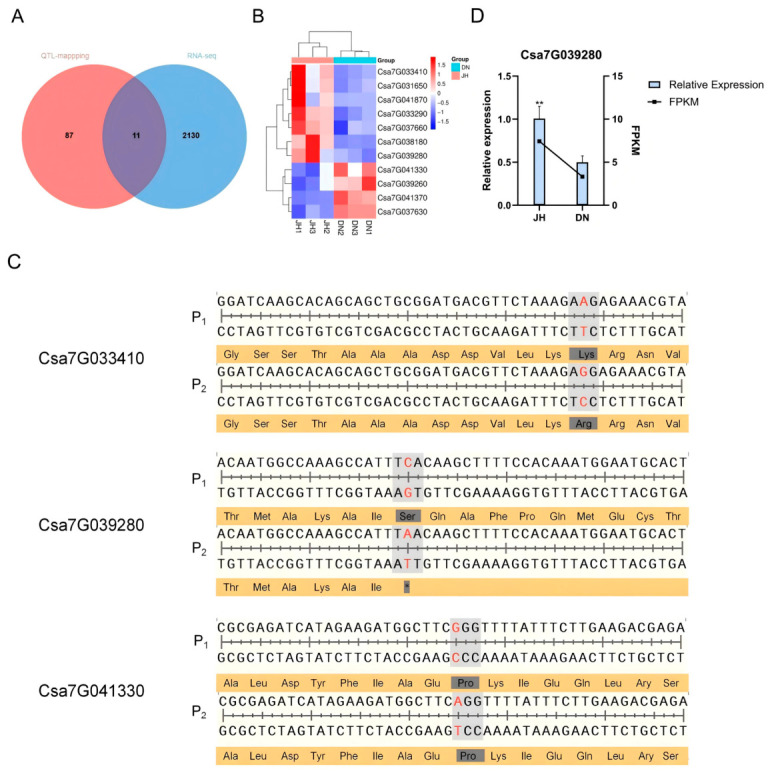
Analysis of candidate genes for hollow hearts in cucumber fruits. (**A**) Identification of candidate genes by integrating genes within QTL intervals and differentially expressed genes; (**B**) Volcano plot displaying the 11 co-expressed candidate genes jointly identified through QTL mapping and RNA-seq analyses; (**C**) Mutation status in coding sequence; (**D**) Validation of the expression levels of the candidate gene, *Csa7G039280*, using qRT-PCR with three biological replicates and three technical replicates. ** indicate significant differences between JH and DN at a significance level of *p* < 0.01. Statistical significance was determined by Student’s *t*-test.

Based on the results of BSA-seq and QTL mapping, a total of 98 genes were identified within the candidate interval K1702765-K2301051. RNA-seq analysis revealed 2141 DEGs between the hollow-heart variety “JZ6-1-2” and the non-hollow-heart variety “D0432-3-4”, showing specific responses to hollow heart formation. By integrating the 2141 DEGs with the QTL mapping interval, 11 co-localized genes were identified (Figure 4A): *Csa7G031650* (Nudix hydrolase), *Csa7G033290* (Calcium-transporting ATPase), *Csa7G033410* (Auxin-induced-like protein), *Csa7G037630* (Transcription initiation factor TFIID subunit), *Csa7G037660* (Translation initiation factor 1A), *Csa7G038180* (Unknown protein), *Csa7G039260* (Fasciclin-like arabinogalactan protein 9.2), *Csa7G039280* (Orcinol O-methyltransferase; contains IPR016461 (Caffeate O-methyltransferase (COMT) family)), *Csa7G041330* (UDP-glycosyltransferase 1), *Csa7G041370* (Knotted-1-like homeobox protein H1), and *Csa7G041870* (Auxin-induced-like protein). Among these, *Csa7G031650*, *Csa7G033290*, *Csa7G033410*, *Csa7G037660*, *Csa7G038180*, *Csa7G039280*, and *Csa7G041870* were upregulated in “JZ6-1-2”, while *Csa7G037630*, *Csa7G039260*, *Csa7G041330*, and *Csa7G041370* were downregulated (Figure 4B).

Sequence comparison between the two parental lines revealed several polymorphisms within the coding regions of the candidate genes. In *Csa7G033410*, a non-synonymous mutation was identified at position 1,888,290 bp, where an A-to-G substitution resulted in the replacement of lysine by arginine. In *Csa7G039280*, a C-to-A substitution at position 2,141,763 bp introduced a premature stop codon (serine to stop), predicted to cause truncation of the translated protein. A synonymous mutation was found in *Csa7G041330* at position 2185330 bp, while no mutations were detected in the coding regions of the other eight candidate genes (Figure 4C). Notably, domain analysis using the NCBI conserved domain database indicated that the premature stop codon in *Csa7G039280* of the paternal line lies within the O-methyltransferase domain, suggesting a likely impact on protein function. Therefore, *Csa7G039280* was identified as a promising candidate gene controlling the hollow-heart trait in cucumber. qRT-PCR analysis revealed that the relative expression level of *Csa7G039280* was significantly higher (*p* < 0.01) in JH compared with DN (Figure 4D).

## 3. Discussion

Fruit hollowness is widely regarded as a physiological disorder characterized by the development of cracks or cavities within the fruit locule. This phenomenon is commonly observed in the fruits of various plants, including plums [19], watermelons [20,21], and peas [22]. In watermelon breeding, the United States Department of Agriculture has named this phenomenon “Hollow heart”, also referred to as internal cracking [23]. In cucumber, hollow heart substantially compromises fruit quality. Affected fruits often exhibit a dry, fibrous texture and reduced juiciness compared to normal crisp fruits, leading to lower consumer acceptability and economic losses for producers. Moreover, the presence of the hollow heart diminishes marketability and may reduce repeat purchases by consumers. In severe cases, extensive cavitation can also negatively impact yield. Investigating the hollow heart trait in cucumber is therefore important not only for improving fruit quality and dietary value but also for enhancing the socio-economic sustainability of cucumber production.

In recent years, the combination of RNA-seq with BSA-seq and QTL mapping has emerged as an effective approach for identifying candidate genes associated with target traits such as stress resistance [24]. This method targets materials with extreme phenotypes, constructs bulked pools, and performs whole-genome resequencing to accurately identify genetic markers such as single-nucleotide polymorphisms (SNPs) and insertions/deletions (InDels) that are closely associated with the target trait, thereby enabling precise localization of the relevant genes. The integration of BSA-seq, QTL mapping, and RNA-seq not only narrows down the candidate regions for genes but also reveals the expression levels of genes within these regions that are related to the target trait. Joint analysis based on the results of these three methods has been widely applied to identify candidate genes for other target traits. For instance, candidate genes regulating potato tuber eye depth and the embryo spot trait have been identified [25,26]; a candidate gene associated with fruit pedicel length in bitter gourd (*Momordica* spp.) [27], along with candidate genes related to photosensitive leaf yellowing in pepper (*Capsicum annuum* L.), has also been identified [28].

BSA-seq analysis initially identified three candidate genomic intervals associated with the hollow-heart trait. The interval on chromosome 2 spanned 0.2 Mb and contained a single gene. Due to the limited size of this interval and the absence of suitable sequence polymorphisms for high-throughput KASP marker development, cosegregation validation in a large population was not performed. Subsequent sequence alignment and expression analysis between the parental lines revealed no differences in either the genomic sequence or expression level of this gene, leading to its exclusion from further consideration. A limitation of this study is the relatively low marker density within the QTL candidate region on chromosome 7, where only eight KASP markers were used to map a 1.7 Mb interval. While this density was sufficient for initial QTL detection in the F_2_ population, it is inadequate for precise fine mapping and candidate gene identification. Future studies employing additional markers, near-isogenic lines, or larger populations will be required to further narrow the candidate interval and validate the causal genes. In addition, the QTL and candidate gene were identified in a single biparental population derived from two specific parental lines. Although these parents represent distinct genetic backgrounds (one hollow and one non-hollow), the general applicability of the findings to other cucumber germplasm remains to be tested.

Genetic improvement for hollow heart resistance is essential to address this constraint, and identifying the key regulatory genes is a critical prerequisite. Although previous studies have made some progress, major knowledge gaps remain: candidate genes have not been functionally verified, the regulatory network underlying hollow heart formation is largely unclear, and most studies have relied on a single mapping or transcriptomic strategy, limiting the integration of genomic variation with gene expression information. In this study, through the integrated application of BSA-seq, QTL mapping, and RNA-seq, *Csa7G039280* was ultimately identified as a probable candidate gene regulating hollow heart formation in cucumber fruit. According to the annotation from the Cucurbitaceae Genomics Database, this gene encodes an Orcinol O-methyltransferase and contains the IPR016461 domain, which belongs to the COMT family. Enzymes of the COMT family are key catalysts in lignin biosynthesis, particularly for S-type lignin. Utilizing S-adenosyl methionine (SAM) as a methyl donor, they catalyze the methylation of various substrates and play important roles in primary and secondary metabolism [29]. Given the highly conserved structure of COMT proteins, it is plausible that these genes perform conserved functions across plant species [30]. In cucumber, comparative transcriptomic studies by Li and Zhang revealed significant differences in lignin content between hollow-heart and non-hollow-heart varieties, suggesting that lignin may influence the hollow-heart phenotype by altering cell wall composition [9,10]. Moreover, it has been reported that lignin is dispersed in the transmitting tract (TT) cells of wild-type plants, whereas in *Csspt Csalc* mutants, lignin accumulates between two cell layers, leading to carpel separation and hollow-heart formation [12]. In the present work, *Csa7G039280* was enriched in secondary metabolite biosynthetic pathways, and its expression level was higher in the hollow-heart variety “JZ6-1-2” than in the non-hollow-heart variety “D0432-3-4”. Therefore, it is hypothesized that *Csa7G039280* may promote lignin synthesis, thereby contributing to the development of hollow heart in cucumber fruit. In future functional validation of *Csa7G039280*, we plan to conduct quantitative analysis of cell-wall components, including lignin content and composition, in the two parental lines. These analyses will help determine whether alterations in lignin metabolism mediated by *Csa7G039280* directly contribute to hollow heart formation. To further validate the role of *Csa7G039280*, gene editing technologies such as CRISPR/Cas9 could be employed to generate knockout mutants, allowing for the observation of whether the hollow-heart phenotype develops in the resulting fruits. Additionally, overexpression constructs could be developed for transgenic complementation assays to confirm the gene’s function. Given that trait regulation in plants often involves gene interactions, yeast one-hybrid and yeast two-hybrid experiments may also be conducted to identify upstream regulators and interacting proteins of the candidate gene. These approaches would help elucidate the molecular pathways involved and further enrich the genetic regulatory network underlying the formation of hollow hearts in cucumber fruit.

## 4. Materials and Methods

### 4.1. Plant Materials and Hollow Heart Evaluation

Two cucumber inbred lines, “JZ6-1-2” and “D0432-3-4”, were used as the female and male parents, respectively. The female parent “JZ6-1-2” is a high-generation inbred line derived from the South China-type cucumber cultivar Jiza 6, which is prone to hollow heart formation in fruit. The male parent “D0432-3-4” is a high-generation inbred line of European greenhouse-type slicing cucumber with no hollow heart trait. A six-generation population was constructed, comprising P_1_ (*n* = 30), P_2_ (*n* = 30), F_1_ (*n* = 40), F_2_ (*n* = 170), BC_1_P_1_ (*n* = 50), and BC_1_P_2_ (*n* = 50). All plants were sown under uniformly controlled conditions in an experimental greenhouse at Acheng Farm, Heilongjiang, China, on 15 April 2022.

Previous studies indicated that fruits of “JZ6-1-2” exhibited typical marketable characteristics and an obvious hollow heart at 9 DPA. Therefore, this stage was determined as the appropriate time for evaluating the hollow heart trait in cucumber. At 9 DPA, five mature fruits with uniform growth vigor were selected from each plant. Each fruit was transversely sectioned at the midpoint, and the cross-section was photographed for phenotypic assessment. The phenotypic evaluation was conducted referring to the cucumber fruit hollow heart evaluation system established by Qin et al., using a 0–4 scale: 0, no hollow heart; 1, slight (0–1% of cross-sectional area); 2, mild (1–10%); 3, moderate (10–40%); 4, severe (≥40%) [18].

### 4.2. Paraffin Sectioning

The carpel suture tissue from cucumber fruits at 9 DPA (indicated by red boxes in Figure 1A), collected from both the hollow-heart variety “JZ6-1-2” and the non-hollow-heart variety “D0432-3-4”, was dissected, cut into 0.5–1 cm^3^ pieces, fixed in formalin-acetic acid-alcohol (FAA) fixative at 4 °C for more than 24 h, then dehydrated through a graded ethanol series and cleared with a xylene-ethanol mixture followed by pure xylene. Subsequently, the samples were embedded in paraffin wax. Sections of appropriate thickness were cut using a microtome (HistoCore AUTOCUT, Leica Microsystems, Wetzlar, Germany). The obtained ribbon sections were deparaffinized, stained, and observed under an OLYMPUS CKX41 inverted microscope (Olympus corporation, Tokyo, Japan). The anatomical structures were analyzed at a scale of 100 μm, and representative fields of view were selected for image capture. The data were processed and analyzed using ImageJ 2023, Excel 2010, and GraphPad Prism v10.1.2.

### 4.3. BSA-Seq and Mapping Analysis

Genomic DNA was extracted from individuals of the P_1_, P_2_, F_1_, and F_2_ populations using an improved CTAB method [31]. Two parental bulked DNA pools (HLP1 and HLP2, each consisting of 25 individuals) were constructed from the inbred lines “D0432-3-4” and “JZ6-1-2”, respectively. Additionally, 25 F_2_ plants with a hollow heart grade of 0 (non-hollow) and 25 with a grade of 3 (hollow) were selected as extreme individuals. Using the same DNA extraction and pooling method, equal amounts of leaf genomic DNA from these selected individuals were mixed to generate the non-hollow (NHL) and hollow (HL) bulks. These four DNA libraries were subjected to whole-genome resequencing on an Illumina HiSeq platform (Biomarker Technologies, Beijing, China). Raw sequencing reads were subjected to quality control. Adapter sequences and low-quality reads were removed, including those in which more than 50% of bases had a Phred quality score (Q) ≤ 10, and paired-end reads with undetermined bases (N) exceeding 10% of their length. Clean reads were retained for downstream analysis. The cleaned resequencing reads were aligned to the Chinese Long v2 reference genome [32] using BWA software (v2.2) [33]. SNP calling was performed using the Genome Analysis Toolkit [34]. Candidate genomic intervals associated with the target trait were then identified by applying the Euclidean distance algorithm to conduct association analysis of SNPs derived from the two bulked samples [35].

### 4.4. QTL Mapping

Based on the BSA-seq results, candidate chromosomal intervals were initially identified. Differential SNP loci between the two parental lines within these regions were subsequently converted into KASP markers by designing two forward competitive primers and one common reverse primer. To further narrow the mapping interval, genotyping was performed on 170 F_2_ individuals using 13 pairs of polymorphic KASP markers (Table S7). Genetic linkage analysis and QTL mapping for the fruit hollow heart trait were conducted using QTL IciMapping v4.1 [36]. Based on the inclusive composite interval mapping method and the Kosambi mapping function [37], QTLs were identified using a step size of 1 cM, a probability in stepwise regression of 0.001, and a LOD (Logarithm of Odds) threshold greater than 2.5, which was determined by 1000 permutation tests at a significance level of α = 0.05 [38].

### 4.5. RNA-Seq and Data Analysis

At 9 DPA, fruit samples were collected from the non-hollow parent “D0432-3-4” (designated DN) and the hollow parent “JZ6-1-2” (designated JH). For each genotype, 0.5 g of tissue was dissected from the carpel suture region, where the three carpels meet and their folded edges fuse (Figure 1A). The dissected tissues were immediately flash-frozen in liquid nitrogen and subsequently stored at −80 °C until further use. Three biological replicates were prepared per sample. Total RNA extraction, cDNA library construction, and RNA sequencing were conducted by Metware Biotechnology (Wuhan, China). After sequencing on a DNB-based high-throughput platform, raw reads were generated and subjected to quality control using fastp [39]. The resulting clean reads were aligned to the reference genome (Chinese Long v2) using HISAT2 (v2.2.1) [40]. Gene expression levels were quantified using FPKM (fragments per kilobase of transcript per million mapped fragments) [41]. In differential expression analysis using the DESeq2 package (v1.22.1) [42,43], the Benjamini–Hochberg method was applied to correct the *p*-values for multiple hypothesis testing, yielding the false discovery rate (FDR). Genes with |log2fold change| ≥ 1 and FDR < 0.05 were considered differentially expressed. GO [44] and KEGG [45] enrichment analyses were performed with the clusterProfiler package (v4.6.0) [46] using the hypergeometric test. GO terms and KEGG pathways with FDR < 0.05 were considered to represent a significant enrichment of DEGs.

### 4.6. Real-Time Quantitative Reverse Transcription-Polymerase Chain Reaction (qRT-PCR) Verification for Sequencing Data

To validate the RNA-seq results, six randomly selected DEGs were subjected to qRT-PCR using the same RNA samples employed for sequencing. qRT-PCR was performed with the Talent qPCR PreMix (SYBR Green, TIANGEN, Beijing, China) in accordance with the manufacturer’s instructions. Each sample included three biological replicates, each run in three technical replicates. β-Actin was used as an internal reference gene (Table S8). Relative expression levels were calculated using the 2^−ΔΔCt^ method. All primers were designed with NCBI Primer-BLAST (http://www.ncbi.nlm.nih.gov/tools/primer-blast/, accessed on 7 August 2025) and are listed in Table S8. Data visualization was conducted using GraphPad Prism v10.1.2.

## 5. Conclusions

In this study, a six-generation population derived from the hollow-heart cucumber variety “JZ6-1-2” and the non-hollow-heart variety “D0432-3-4” was used as experimental material. After conducting BSA-seq, candidate regions for the hollow-heart trait in cucumber fruits were identified on chromosomes 2, 3, and 7. Subsequently, using KASP molecular markers, a major QTL associated with the hollow-heart trait was mapped on chromosome 7, located between K1702765 and K2301051, spanning approximately 0.6 Mb and containing 98 genes. Based on RNA-seq analysis, 2141 DEGs related to the hollow-heart trait were identified. Among these, 11 genes were found to overlap with the QTL region. Further, through qRT-PCR, gene sequence comparison, and gene annotation analysis, *Csa7G039280* was predicted as a key candidate gene regulating hollow-heart formation in cucumber fruits, although further functional studies are required to confirm its role.

## Figures and Tables

**Table 1:** Phenotypic characteristic of hollow heart in six generation.

Generation	Hollow Grade	Max Grade	Min Grade	Kurtosis	Skewness
P_1_ (*n* = 30)	3 ± 0	3	3	-	-
P_2_ (*n* = 30)	0	0	0	-	-
F_1_ (*n* = 40)	1.31 ± 0.32	1.78	0.8	-	-
F_2_ (*n* = 170)	0.74 ± 0.61	3	0	1.231	1.05
BC_1_P_1_ (*n* = 50)	0.88 ± 0.9	3.33	0	0.99	1.16
BC_1_P_2_ (*n* = 50)	0.37 ± 0.46	1.8	0	0.77	1.15

The hollow grade was scored on a 0–4 scale based on the severity of cavity formation: 0, no hollow heart; 1, slight (0–1% of cross-sectional area); 2, mild (1–10%); 3, moderate (10–40%); 4, severe (≥40%). Hollow grade values are presented as mean ± standard deviation (SD). -, not applicable.

## Data Availability

Data are contained within the article and Supplementary Materials.

## Supplementary Materials

The following supporting information can be downloaded at: https://www.mdpi.com/article/10.3390/plants15091299/s1, Figure S1: Cucumber genetic linkage map and QTL mapping results; Figure S2: qRT-PCR verification for transcriptome data; Table S1: Statistics of sequencing data; Table S2: Statistics of reference genome alignment; Table S3: Statistics of sequencing depth and genome coverage; Table S4. Distribution of QTL on the genetic linkage map; Table S5: Genes within the QTL-mapped regions; Table S6: RNA-seq data statistics; Table S7: KASP marker names and sequence; Table S8: Primer sequences for reference gene and target genes used in qRT-PCR assays.
