# Supplementary material for: Integrating BSA-Seq, QTL Mapping, and RNA-Seq to Identify Candidate Genes for Hollow Heart in Cucumber Fruits
Source: Plants (Basel). 2026 Apr 23;15(9):1299. doi: 10.3390/plants15091299 (PMC13165383; doi:10.3390/plants15091299)
Supplement: Supplementary file 1 [file plants-15-01299-s001.zip › Supplementary Figure.pdf]

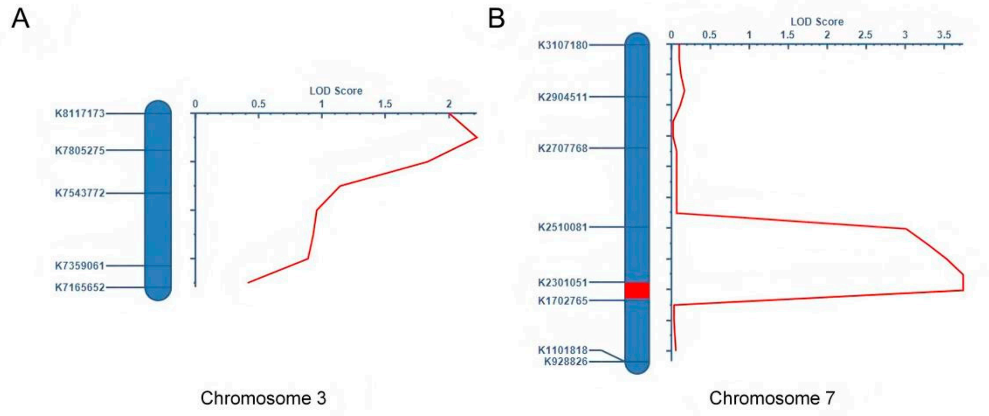

Figure S1. Cucumber genetic linkage map and QTL mapping results. The red-marked section indicates the QTL interval. The horizontal axis represents the QTL LOD score, and the vertical axis represents the genetic distance between the inserted markers. (A) Linkage map and QTL mapping on chromosome 3; (B) Linkage map and QTL mapping on chromosome 7.

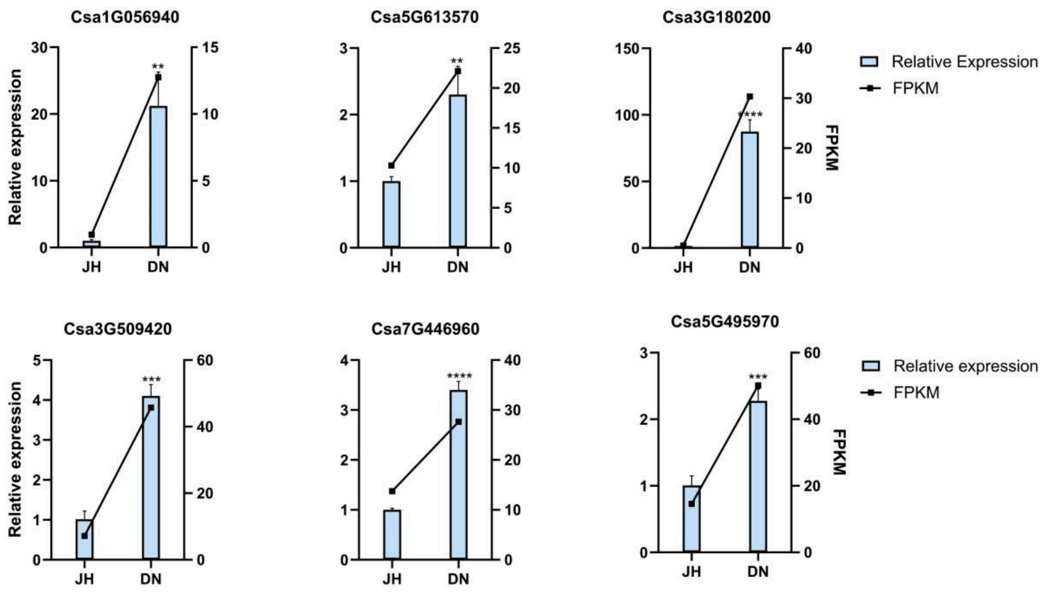

Figure S2. qRT-PCR verification for transcriptome data. Each sample included three biological replicates, each run in three technical replicates. FPKM, fragments per kilobase of the exon model per million mapped fragments. **, ***, and **** indicate significant differences between JH and DN at significance levels of $p<0.01$, $p<0.001$, and $p<0.0001$ respectively. Statistical significance was determined by Student's t-test.
