# Supplementary material for: Integrating BSA-Seq, QTL Mapping, and RNA-Seq to Identify Candidate Genes for Hollow Heart in Cucumber Fruits
Source: Plants (Basel). 2026 Apr 23;15(9):1299. doi: 10.3390/plants15091299 (PMC13165383; doi:10.3390/plants15091299)
Supplement: Supplementary file 1 [file plants-15-01299-s001.zip › SupplementaryTable.pdf]

Table S1. Statistics of sequencing data

Bulked pool	Clean reads	Clean base	Q30 (%)	GC (%)
HLP1	18,893,851	5,617,850,280	94.58	36.28
HLP2	20,665,783	6,138,047,364	95.09	37.06
HL	28,407,580	28,407,580	94.55	36.63
NHL	27,484,552	27,484,552	94.83	36.88

Table S2. Statistics of reference genome alignment

Bulked pool	Total reads	Mapped reads (%)	Properly mapped reads(%)
HL	56,815,160	92.12	73.39
HLP1	37,787,702	94.03	76.42
HLP2	41,331,566	94.04	75.92
NHL	54,969,104	93.10	74.64

Note: Total Reads, the total number of clean reads in the sample, counted separately for paired ends (i.e., read1 and read2 are counted as two reads). Mapped reads(%), the percentage of clean reads that are aligned to the reference genome out of the total clean reads. Properly Mapped reads(%), the percentage of paired-end reads where both ends are aligned to the reference genome with a distance consistent with the expected fragment length distribution.

Table S3. Statistics of sequencing depth and genome coverage

Bulked pool	Average depth	Covariance Ratio _1×(%)	Covariance Ratio_5×(%)	Covariance Ratio_10×(%)
HL	37	99.45	98.58	95.04
HLP1	25	99.12	97.39	86.89
HLP2	27	99.23	97.51	87.29
NHL	36	99.45	98.58	94.88

Table S4. Distribution of QTL on the genetic linkage map

Chromosome	LOD	PVE（%）	Additive	Dominant	Left Marker	Right Marker
7	3.7432	9.5269	-0.2690	-0.0482	K2301051	K1702765

Note: PVE（%）, phenotypic variation explained.

Table S5. Genes within the QTL-mapped regions

Gene ID	Description	Location (chromosome 7)
Csa7G031600	Long-chain-fatty-acid CoA ligase, putative; contains IPR000873 (AMP-dependent synthetase/ligase)	1711103 .. 1717161 (+)
Csa7G031610	Putative methyltransferase; contains IPR011192 (Rubisco methyltransferase)	1719689 .. 1725122 (+)
Csa7G031620	Indole-3-glycerol phosphate synthase; contains IPR013785 (Aldolase-type TIM barrel)	1727461 .. 1731799 (+)
Csa7G031630	Transmembrane emp24 domain-containing protein; contains IPR009038 (GOLD)	1734757 .. 1741229 (+)
Csa7G031640	3-ketoacyl-CoA thiolase; contains IPR002155 (Thiolase), IPR016039 (Thiolase-like)	1742984..1745352 (-)
Csa7G031650	Nudix hydrolase; contains IPR003293 (Nudix hydrolase 6-like)	1757834 .. 1761505 (+)
Csa7G031660	Fasciclin-like arabinogalactan protein 19; contains IPR000782 (FAS1 domain)	1761901..1764488 (-)
Csa7G031670	Unknown protein	1772725..1772987 (-)
Csa7G031680	Unknown protein	1773366..1773776 (-)
Csa7G031690	5'-AMP-activated protein kinase subunit beta; contains IPR006828 (5-AMP-activated protein kinase, beta subunit, interaction domain), IPR014756 (Immunoglobulin E-set)	1774407..1776728 (+)
Csa7G031700	Alpha-1,6-mannosyl-glycoprotein 2-beta-N-acetylglucosaminyltransferase; contains IPR007754 (N-acetylglucosaminyltransferase II)	1782728..1784991 (+)
Csa7G031710	Unknown protein	1785194..1785683 (+)
Csa7G031720	Phytochrome; contains IPR001294 (Phytochrome)	1788632..1794057 (+)
Csa7G031730	Pentatricopeptide repeat-containing protein; contains IPR002885 (Pentatricopeptide repeat), IPR011990 (Tetratricopeptide-like helical)	1794858..1798206 (+)
Csa7G031740	FAR1-related protein; contains IPR004330 (FAR1 DNA binding domain)	1799957..1802915 (+)
Csa7G032240	Unknown protein	1807424..1808038 (-)
Csa7G032250	Unknown protein	1813279..1813697 (+)
Csa7G032260	Receptor-like protein kinase; contains IPR001611 (Leucine-rich repeat)	1814535..1815571 (+)
Csa7G032270	Chaperone protein dnaJ; contains IPR001623 (DnaJ domain), IPR024593 (Domain of unknown function DUF3444)	1819347..1823445 (+)
Csa7G032280	CDO504; contains IPR006696 (Protein of unknown function DUF423)	1823539..1825653 (+)
Csa7G032290	Nup133 nucleoporin family protein, expressed; contains IPR007187 (Nucleoporin,	1826730..1833225 (+)

	Nup133/Nup155-like, C-terminal), IPR014908 (Nucleoporin, Nup133/Nup155-like, N-terminal)	
Csa7G032790	Unknown protein	1840988..1841557 (+)
Csa7G033290	Calcium-transporting ATPase; contains IPR001757 (Cation-transporting P-type ATPase), IPR023214 (HAD-like domain), IPR023298 (P-type ATPase, transmembrane domain)	1843440..1846215 (+)
Csa7G033300	Chlorophyll a-b-binding protein 5, chloroplastic; contains IPR022796 (Chlorophyll A-B binding protein), IPR023329 (Chlorophyll a/b binding protein domain)	1847096..1848898 (+)
Csa7G033310	Pentatricopeptide repeat-containing protein; contains IPR002885 (Pentatricopeptide repeat), IPR011990 (Tetratricopeptide-like helical)	1848695..1850405 (-)
Csa7G033320	Unknown protein	1850723..1851318 (-)
Csa7G033330	Potyviral VPg interacting protein 1; contains IPR004082 (Protein of unknown function DUF1423, plant)	1853590..1858239 (+)
Csa7G033340	Fasciclin-like arabinogalactan family protein; contains IPR000782 (FAS1 domain)	1857673..1858266 (-)
Csa7G033350	WRKY transcription factor 2; contains IPR003657 (DNA-binding WRKY)	1861875..1863857 (-)
Csa7G033360	Unknown protein	1863880..1865315 (-)
Csa7G033370	Protein DGCR14; contains IPR019148 (Nuclear protein DGCR14)	1868796..1870683 (+)
Csa7G033380	BTB/POZ domain-containing protein; contains IPR011333 (BTB/POZ fold), IPR027356 (NPH3 domain)	1871777..1874422 (+)
Csa7G033390	Auxin induced-like protein; contains IPR004877 (Cytochrome b561, eukaryote), IPR005018 (DOMON domain), IPR017214 (Uncharacterised conserved protein UCP037471)	1877257..1878923 (-)
Csa7G033400	N-acetyltransferase 9-like protein; contains IPR016181 (Acyl-CoA N-acyltransferase)	1880288..1883047 (+)
Csa7G033410	Auxin induced-like protein; contains IPR004877 (Cytochrome b561, eukaryote), IPR005018 (DOMON domain), IPR017214 (Uncharacterised conserved protein UCP037471)	1887471..1889171 (+)
Csa7G033420	Calcium-binding protein, putative; contains IPR013878 (Mo25-like)	1891559..1894838 (-)
Csa7G033430	Threonine--tRNA ligase; contains IPR002320 (Threonine-tRNA ligase, class IIa)	1897552..1902148 (+)
Csa7G033440	Polygalacturonase; contains IPR000743 (Glycoside hydrolase, family 28), IPR011050 (Pectin lyase	1902943..1905210 (+)

	fold/virulence factor)	
Csa7G033940	Polygalacturonase 3; contains IPR000743 (Glycoside hydrolase, family 28), IPR011050 (Pectin lyase fold/virulence factor)	1908675..1910485 (+)
Csa7G035440	Unknown protein	1916326..1916738 (-)
Csa7G036440	Unknown protein	1924657..1924990 (-)
Csa7G036450	Callose synthase 5	1928641..1928896 (+)
Csa7G037450	Polygalacturonase; contains IPR000743 (Glycoside hydrolase, family 28), IPR008238 (Chorismate mutase, AroQ class, eukaryotic type), IPR011050 (Pectin lyase fold/virulence factor)	1932935..1934065 (+)
Csa7G037460	Polygalacturonase; contains IPR000743 (Glycoside hydrolase, family 28), IPR011050 (Pectin lyase fold/virulence factor)	1936575..1937720 (+)
Csa7G037470	UDP-glycosyltransferase 1; contains IPR002213 (UDP-glucuronosyl/UDP-glucosyltransferase)	1940261..1941864 (+)
Csa7G037480	Unknown protein	1945220..1947872 (-)
Csa7G037500	Rho GDP-dissociation inhibitor, putative; contains IPR000406 (RHO protein GDP dissociation inhibitor), IPR014756 (Immunoglobulin E-set)	1962323..1965088 (+)
Csa7G037510	Fasciclin-like arabinogalactan protein 12.2; contains IPR000782 (FAS1 domain)	1968895..1970635 (+)
Csa7G037520	Fimbrin, putative; contains IPR001715 (Calponin homology domain)	1972739..1978504 (+)
Csa7G037530	Unknown protein	1980489..1980832 (-)
Csa7G037540	Unknown protein	1990087..1991138 (-)
Csa7G037550	Unknown protein	1994081..1994553 (-)
Csa7G037560	Glycine-rich protein	2006009..2006896 (-)
Csa7G037570	Polygalacturonase; contains IPR000743 (Glycoside hydrolase, family 28), IPR011050 (Pectin lyase fold/virulence factor)	2007896..2008791 (-)
Csa7G037580	Unknown protein	2010684..2010936 (-)
Csa7G037590	Unknown protein	2012733..2013778 (-)
Csa7G037600	Cell wall glycine-rich protein; contains IPR010800 (Glycine rich protein)	2021483..2022548 (-)
Csa7G037610	Pathogen-induced protein CuPi1	2037564..2038288 (-)
Csa7G037620	Calcium-binding EF-hand family protein-like; contains IPR011992 (EF-hand-like domain)	2046439..2046972 (+)
Csa7G037630	Transcription initiation factor TFIID subunit; contains IPR009072 (Histone-fold), IPR011442 (Domain of unknown function DUF1546), IPR016024 (Armadillo-type fold)	2050310..2057165 (-)
Csa7G037640	Unknown protein	2057183..2058216 (-)
Csa7G037650	IQ domain-containing protein; contains IPR000048	2060615..2062922 (+)

	(IQ motif, EF-hand binding site)	
Csa7G037660	Translation initiation factor 1A; contains IPR001253 (Translation initiation factor 1A (eIF-1A))	2063391..2065086 (-)
Csa7G038160	OB-fold nucleic acid binding domain containing protein; contains IPR012340 (Nucleic acid-binding, OB-fold)	2070530..2072515 (+)
Csa7G038170	Unknown protein	2072751..2073156 (-)
Csa7G038180	Unknown protein	2073213..2073986 (-)
Csa7G038680	DDB1-and CUL4-associated factor; contains IPR007287 (Sof1-like protein), IPR015943 (WD40/YVTN repeat-like-containing domain), IPR020472 (G-protein beta WD-40 repeat)	2074628..2079705 (-)
Csa7G038690	26S proteasome regulatory subunit N1; contains IPR016643 (26S proteasome regulatory complex, non-ATPase subcomplex, Rpn1 subunit)	2080654..2089143 (-)
Csa7G038700	TED3	2090985..2092001 (-)
Csa7G039200	Chaperone protein dnaJ; contains IPR001623 (DnaJ domain), IPR002939 (Chaperone DnaJ, C-terminal)	2096427..2098557 (+)
Csa7G039210	Unknown protein	2098930..2099235 (-)
Csa7G039220	Unknown protein	2099587..2100134 (+)
Csa7G039230	Receptor kinase; contains IPR001611 (Leucine-rich repeat), IPR003591 (Leucine-rich repeat, typical subtype), IPR011009 (Protein kinase-like domain), IPR013210 (Leucine-rich repeat-containing N-terminal, type 2), IPR025875 (Leucine rich repeat 4)	2106445..2110085 (+)
Csa7G039240	Zinc finger family protein; contains IPR013087 (Zinc finger C2H2-type/integrase DNA-binding domain)	2113710..2117275 (-)
Csa7G039250	Lysine-specific histone demethylase-like protein	2122640..2126111 (+)
Csa7G039260	Fasciclin-like arabinogalactan protein 9.2; contains IPR000782 (FAS1 domain)	2129821..2131642 (-)
Csa7G039270	AT3g54680/T5N23_40	2133927..2137164 (+)
Csa7G039280	Orcinol O-methyltransferase; contains IPR016461 (Caffeate O-methyltransferase (COMT) family)	2140976..2142654 (+)
Csa7G039780	Putative orcinol O-methyltransferase; contains IPR016461 (Caffeate O-methyltransferase (COMT) family)	2148350..2151769 (-)
Csa7G039790	Orcinol O-methyltransferase-like protein; contains IPR016461 (Caffeate O-methyltransferase (COMT) family)	2157962..2159425 (-)
Csa7G041290	Optic atrophy 3 protein, putative; contains IPR010754 (Optic atrophy 3-like)	2167640..2169814 (+)
Csa7G041300	UDP-glycosyltransferase 1; contains IPR002213 (UDP-glucuronosyl/UDP-glucosyltransferase)	2170698..2172080 (-)

Csa7G041310	Pentatricopeptide repeat-containing protein; contains IPR002885 (Pentatricopeptide repeat)	2174150..2175460 (-)
Csa7G041320	UDP-glycosyltransferase 1; contains IPR002213 (UDP-glucuronosyl/UDP-glucosyltransferase)	2180189..2182336 (-)
Csa7G041330	UDP-glycosyltransferase 1; contains IPR002213 (UDP-glucuronosyl/UDP-glucosyltransferase)	2183971..2186095 (-)
Csa7G041340	GTP binding/translation elongation factor protein; contains IPR005225 (Small GTP-binding protein domain), IPR006298 (GTP-binding protein TypA), IPR009000 (Translation elongation/initiation factor/Ribosomal, beta-barrel), IPR027417 (P-loop containing nucleoside triphosphate hydrolase)	2193014..2200374 (-)
Csa7G041350	Glucosidase II beta subunit, putative	2201722..2204700 (+)
Csa7G041360	BZIP transcription factor family protein; contains IPR004827 (Basic-leucine zipper domain)	2206497..2209767 (+)
Csa7G041370	Knotted-1-like homeobox protein H1; contains IPR005539 (ELK), IPR005540 (KNOX1), IPR005541 (KNOX2), IPR009057 (Homeodomain-like)	2231073..2235700 (+)
Csa7G041870	Auxin induced-like protein; contains IPR004877 (Cytochrome b561, eukaryote), IPR005018 (DOMON domain), IPR017214 (Uncharacterised conserved protein UCP037471)	2244154..2245558 (+)
Csa7G041880	BAH-PHD domain-containing protein; contains IPR001025 (Bromo adjacent homology (BAH) domain), IPR013083 (Zinc finger, RING/FYVE/PHD-type)	2250786..2254559 (+)
Csa7G041890	AP-2 complex subunit beta-1, putative; contains IPR009028 (Coatomer/calthrin adaptor appendage, C-terminal subdomain), IPR012295 (Beta2-adaptin/TBP, C-terminal domain), IPR013041 (Coatomer/clathrin adaptor appendage, Ig-like subdomain), IPR015151 (Beta-adaptin appendage, C-terminal subdomain), IPR016024 (Armadillo-type fold), IPR026739 (AP complex subunit beta)	2256217..2262114 (-)
Csa7G041900	Enzyme of the cupin superfamily; contains IPR014710 (RmlC-like jelly roll fold)	2262693..2264394 (-)
Csa7G041910	FAD dependent oxidoreductase	2264472..2270144 (+)
Csa7G041920	Unknown protein	2269828..2272068 (-)
Csa7G041930	Receptor protein kinase, putative; contains IPR011009 (Protein kinase-like domain), IPR013320 (Concanavalin A-like lectin/glucanase, subgroup)	2283047..2287969 (+)
Csa7G041940	Ribonuclease 3-like protein; contains IPR000999 (Ribonuclease III domain), IPR014720 (Double-stranded RNA-binding-like domain)	2291905..2294380 (+)

Csa7G041950	Ubiquitin fusion degradation protein-like protein; contains IPR004854 (Ubiquitin fusion degradation protein UFD1)	2295442..2299100 (+)

Table S6. RNA-seq data statistics

Sample	Raw reads	Clean reads	Q30(%)	GC cntent(%)	Mapping rate
DN1	56847220	56153624	97.5	44.89	98.29%
DN2	66351250	65460590	97.59	45.54	98.24%
DN3	70984508	69990742	97.67	45.13	98.18%
JH1	102941516	101525906	97.91	44.1	98.23%
JH2	73571390	72502732	97.79	44.9	98.10%
JH3	74903696	73613382	98.17	45.18	97.96%

Table S7. KASP marker names and sequence

Maker	Forward primer	Resver primer
K7165652	GAAGGTGACCAAGTTCATGCTAGCAGAACTTGCTTCCAGATCTC<br>GAAGGTCGGAGTCAACGGATTCAGCAGAACTTGCTTCCAGATCTT	TACCTGAAAGTTCAAAGTGGAAGTCAAG
K7359061	GAAGGTGACCAAGTTCATGCTATACCTTGGGTCGATCCATAGAG<br>GAAGGTCGGAGTCAACGGATTTAAATACCTTGGGTCGATCCATAGAA	TTCCCTTTCGTTTGGAATGGGCTAC
K7543772	GAAGGTGACCAAGTTCATGCTAGGAACCCTCAACATGTAGAAATCAA<br>GAAGGTCGGAGTCAACGGATTAGGAACCCTCAACATGTAGAAATCAT	GGGAGATTTTGTATTTGAATCATTTTCTCTC
K7805275	GAAGGTGACCAAGTTCATGCTACAATAAATTTTGGCTGCCCACCATA<br>GAAGGTCGGAGTCAACGGATTACAATAAATTTTGGCTGCCCACCATT	CACGGACGAAGGGAGACTATATTTC
K8117173	GAAGGTGACCAAGTTCATGCTTAAAAGCTTATAGTCATTGTTTACACAAATG<br>GAAGGTCGGAGTCAACGGATTTAAAAGCTTATAGTCATTGTTTACACAAATC	CTCCATTCCCTTACTTTACCATAACTAAC
K9288826	GAAGGTGACCAAGTTCATGCTAAACAGTATTGAATATTTCCATAGTAATATAATTTA<br>GAAGGTCGGAGTCAACGGATTACAGTATTGAATATTTCCATAGTAATATAATTTC	AACTCTCTTAAATAAATACAAACATGGTAGGAAA
K1101818	GAAGGTGACCAAGTTCATGCTACAAAGAAAATCAAACACAACATAAAACTG<br>GAAGGTCGGAGTCAACGGATTACAAAGAAAATCAAACACAACATAAAACTC	GTTTATTTCTTAATTGTACTATTTGTTATAATTGTCC
K1702765	GAAGGTGACCAAGTTCATGCTATAACGAGCCACTCTTCTCAATTGA<br>GAAGGTCGGAGTCAACGGATTAACGAGCCACTCTTCTCAATTGC	TGGACCTGGAAGAGAGAATGGAGTA
K2301051	GAAGGTGACCAAGTTCATGCTAATGAATAGAAACAAATTAAAGTGTGATGTG<br>GAAGGTCGGAGTCAACGGATTTTAAATGAATAGAAACAAATTAAAGTGTGATGTA	TTTAAAATATCAAGGACACTTCCAATCAATTTTC
K2510081	GAAGGTGACCAAGTTCATGCTCTCTCACGACTAGTGATGTTGGTT<br>GAAGGTCGGAGTCAACGGATTTCTCACGACTAGTGATGTTGGTG	TGTGTAGCTTGAGGTTATTACCAATAATAAG
K2707768	GAAGGTGACCAAGTTCATGCTTGGCGGTTTGTTAACTTCCAAAACAT	AGACCATCAATGAGTAATATTGTCCAAATTC

	GAAGGTCGGAGTCAACGGATTGGCGGTTTGTTAACTTCCAAAACAC	
K2904511	GAAGGTGACCAAGTTCATGCTTTAACAAATTTGACAAAATATTTGCAACATATAC<br>GAAGGTCGGAGTCAACGGATTAATTAACAAATTTGACAAAATATTTGCAACATATAT	AATTCTATCAACTATATATATAGAAATCGATACAATTTG
K3107180	GAAGGTGACCAAGTTCATGCTGTACACAATTACTTTTCGTTTCCTTTTG<br>GAAGGTCGGAGTCAACGGATTCGTACACAATTACTTTTCGTTTCCTTTTA	CTTCGAAACCGAAGAAAATTGGGAATAC

Table S8. Primer sequences for reference gene and target genes used in qRT-PCR assays

Gene	Forward primer	Resver primer
β-Actin	TCGTGCTGGATTCTGGTG	GGCAGTGGTGGTGAACAT
Csa1G056940	AGAGAGATTGCGTTTGGGCT	CCGTGCGACTTAGAAACCCT
Csa5G613570	TCAAGTAAGGGGCGGAGAGA	AGGACCAAACTGCCGGAAAA
Csa3G180200	ACCCCGTGATGTTAGAGTTCA	CTTTTAACGACATAGTGTGCCAA
Csa3G509420	TTGATGGGGTTGGACTTGGG	AAATGGCGTTCGATCCCTGA
Csa7G446960	ACAGTGCTTCTGCCTCATGT	ACGAGATCTATGATAACAAGGAGTG
Csa5G495970	GGAAAGGTACAGGCGACACA	CTCGCTGCTTCTGTTGATGC
Csa7G039280	ACTTTCCATGCTCCAGCCAG	TGTACTTCCAGAAGGGCACTC
